# Supplementary material for: Comparing Super-Resolution Microscopy Techniques to Analyze Chromosomes
Source: Int J Mol Sci. 2021 Feb 14;22(4):1903. doi: 10.3390/ijms22041903 (PMC7917581; doi:10.3390/ijms22041903)
Supplement: Supplementary file 1 [file ijms-22-01903-s001.pdf]

Supplementary data

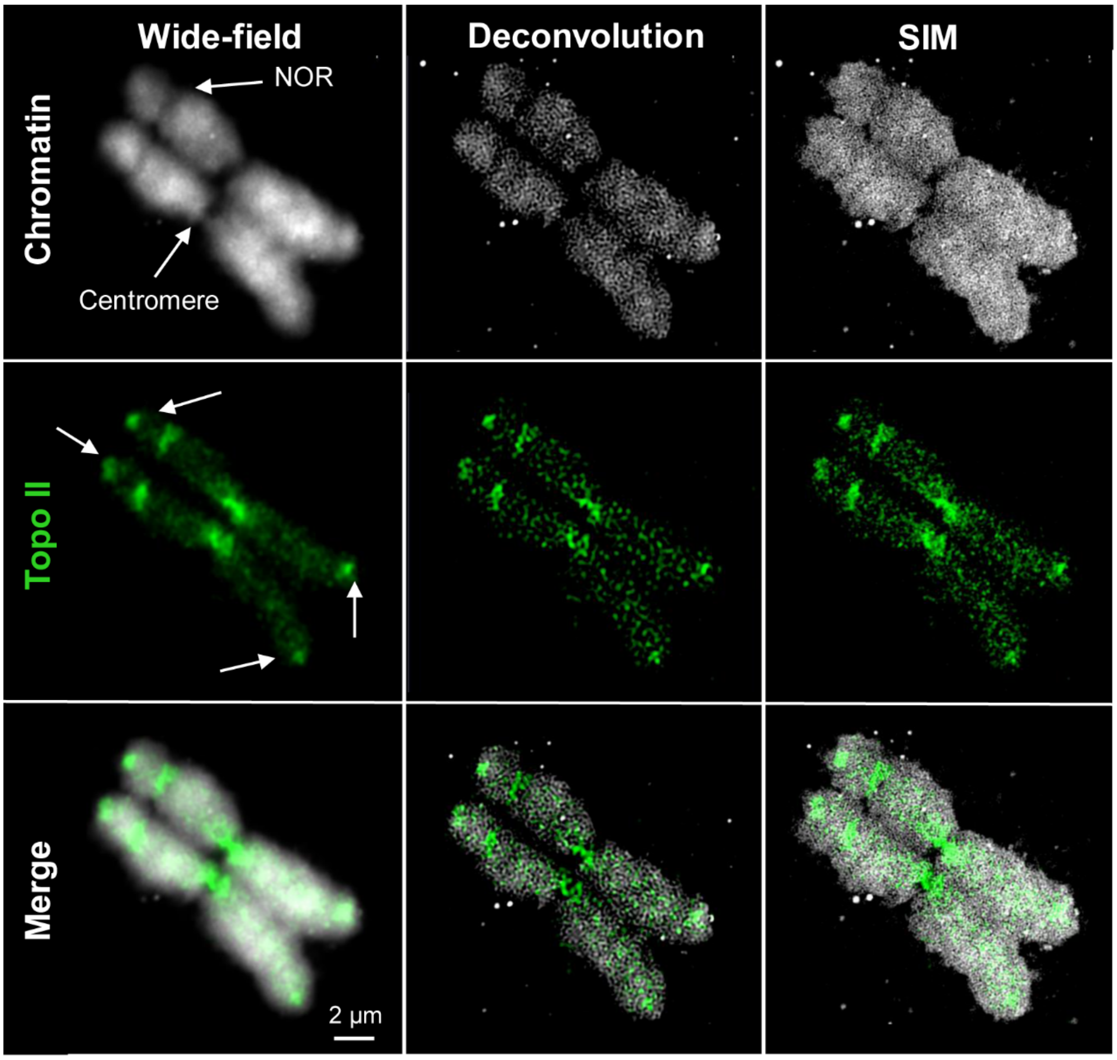

Figure S1. Distribution of Topo II in the barley metaphase satellite chromosome 6H carrying a secondary constriction (nucleolus organizer region – NOR) visualized by wide-field, deconvolution, and structured illumination microscopy (SIM) using Alexa488-labeled secondary antibodies. Topo II is accumulated at the centromere, NOR, and subtelomeres (arrows).

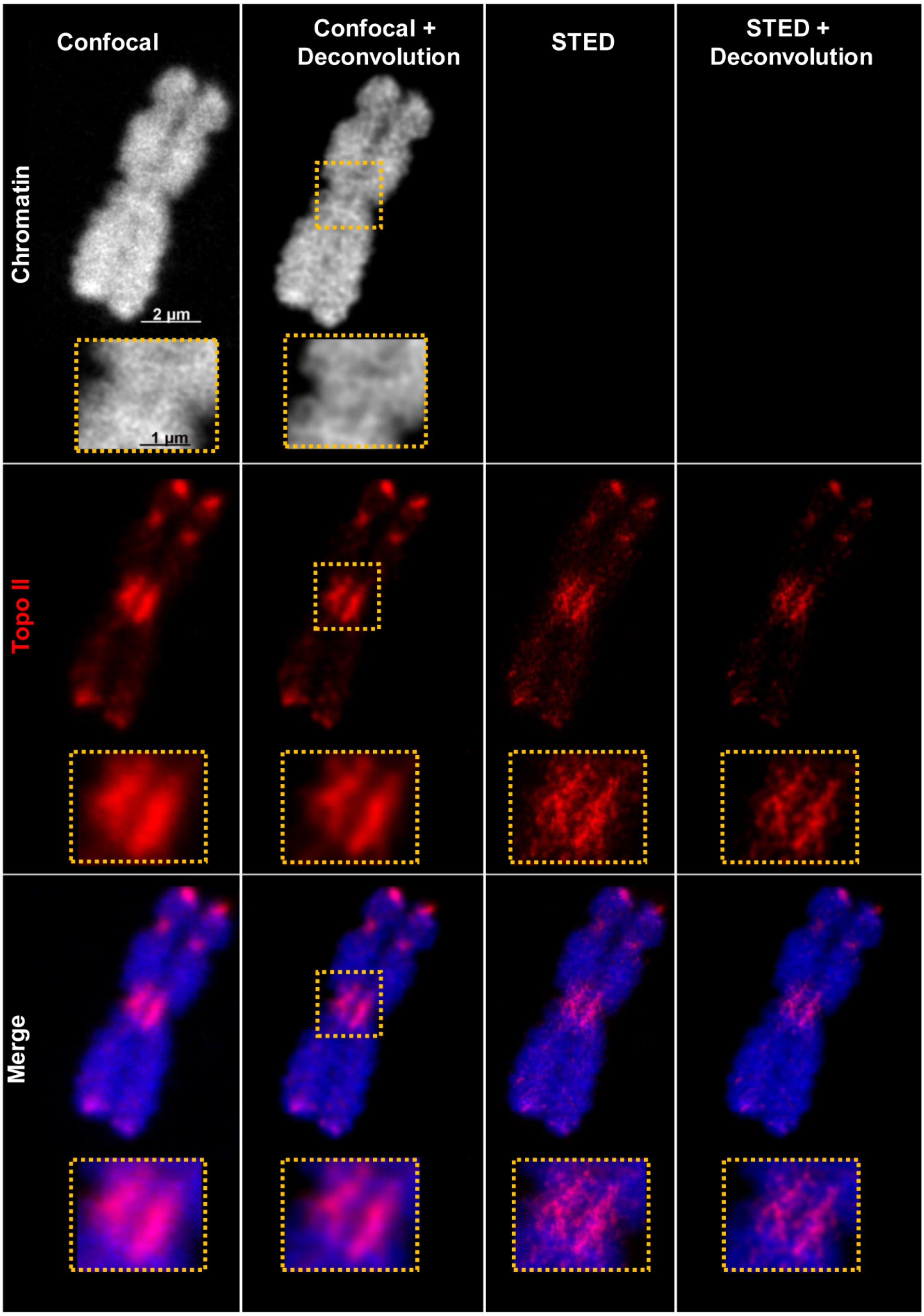

Figure S2. Distribution of Topo II in a barley metaphase satellite chromosome 6H visualized by confocal, confocal+deconvolution, and stimulated emission depletion (STED)+deconvolution using STAR635P-labeled secondary antibodies designated for STED. The enlarged regions (dashed rectangles) show clearly the improved resolution achieved via STED. The STED images were merged with confocal DAPI-labeled chromatin images (blue).

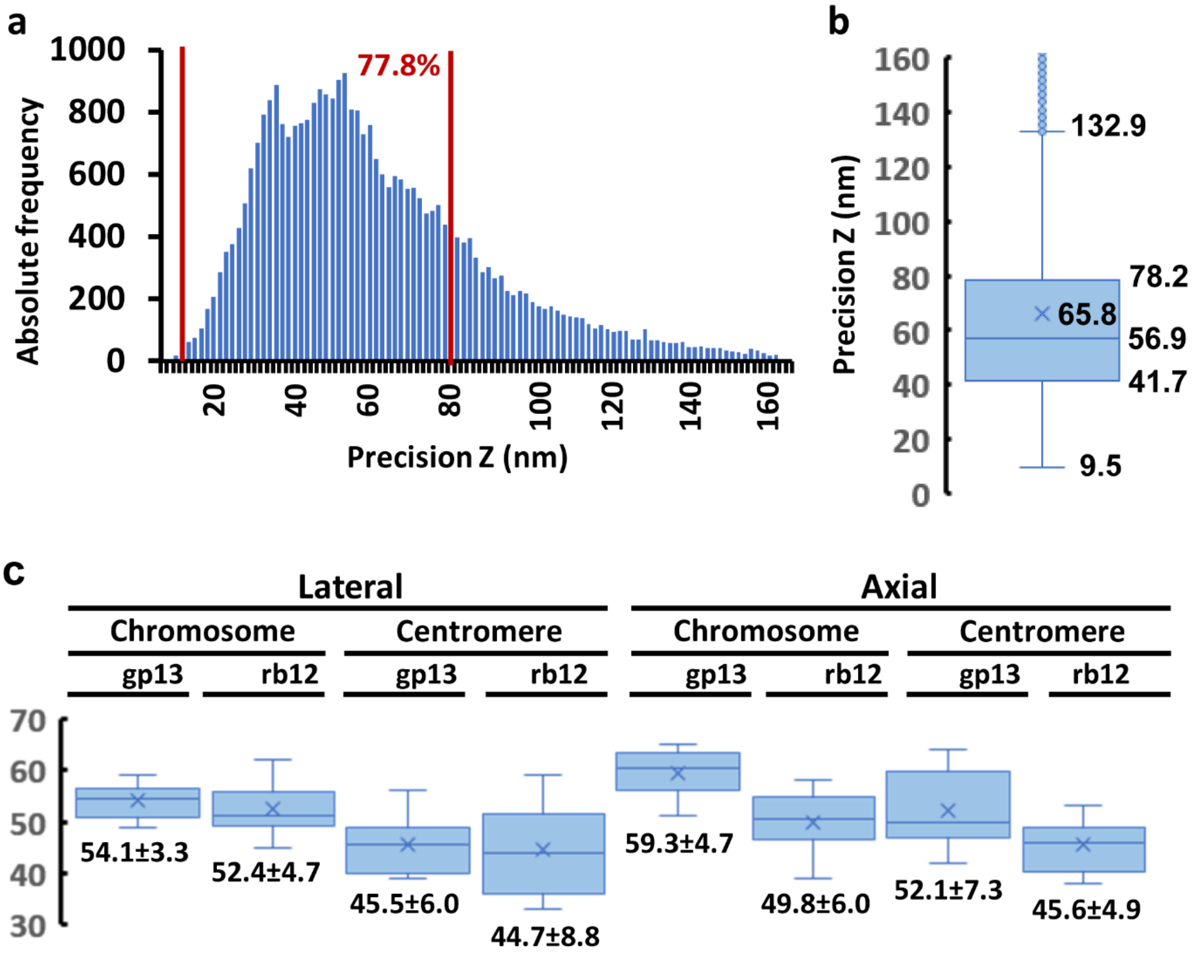

a
1000
800
600
400
200
0
Absolute frequency
77.8%
20
40
60
80
100
120
140
160
Precision Z (nm)
b
160
140
120
100
80
60
40
20
0
Precision Z (nm)
132.9
78.2
65.8
56.9
41.7
9.5
c
Lateral
Axial
Chromosome
Centromere
Chromosome
Centromere
gp13
rb12
gp13
rb12
gp13
rb12
gp13
rb12
70
60
50
40
30
54.1±3.3
52.4±4.7
45.5±6.0
44.7±8.8
59.3±4.7
49.8±6.0
52.1±7.3
45.6±4.9

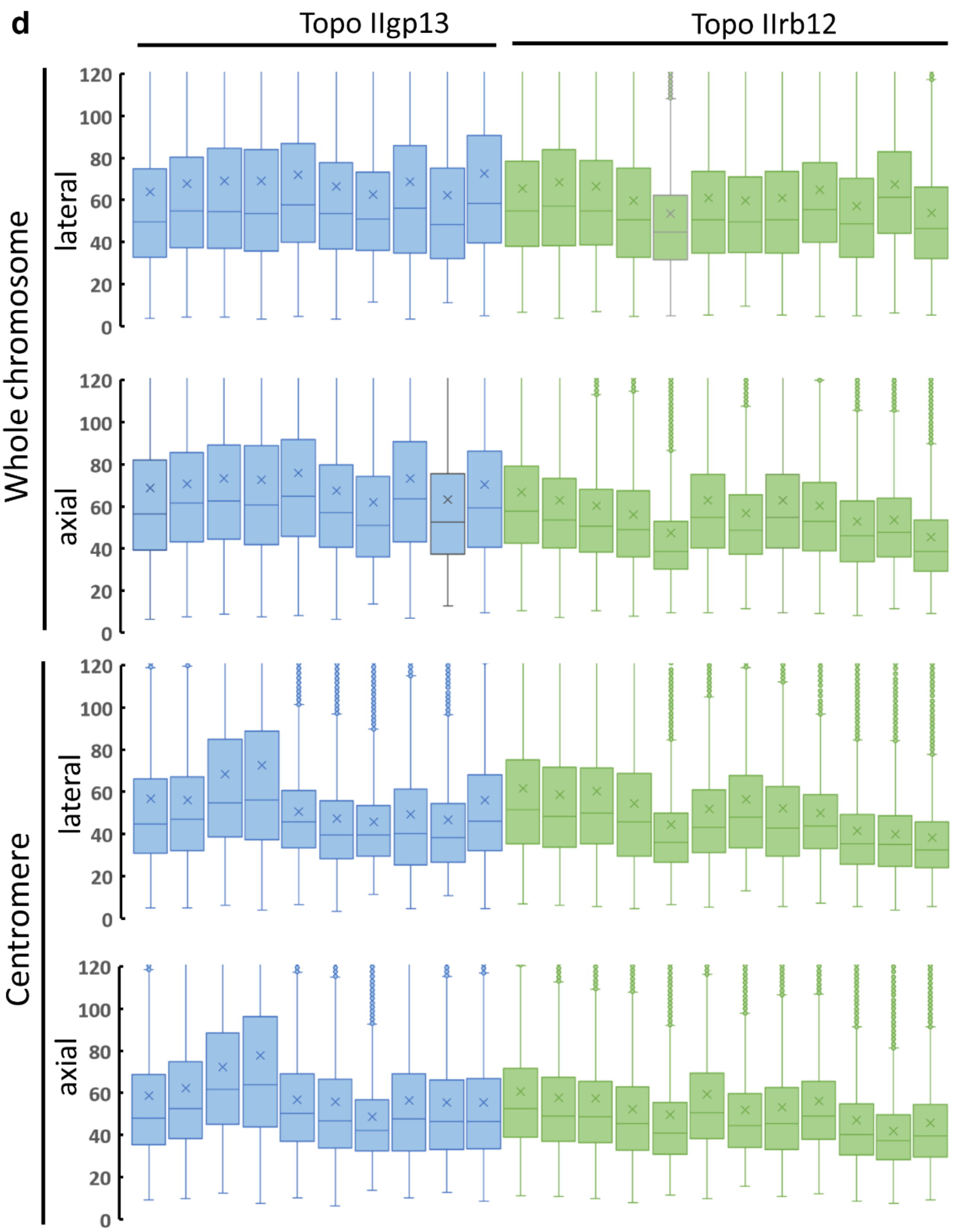

Figure S3. Comparison of axial and lateral precisions achieved along chromosome arms and within the pericentromeres to detect single Topo II molecules labeled by different antibodies (gp13 and rb12). (a) Diagram showing the 3D-PALM axial-Z-localization precision of all Topo IIrb12 labeled molecules detected in the barley chromosome shown in Figure 4. The red bars frame the 77.8% of molecules that were localized with a precision of 10-80 nm. **(b)** Distribution of the same Z localization precisions displayed as a boxplot. Numbers indicate lower whisker (9.5 nm), 25% quantile (41.4 nm), median (56.9 nm), mean (65.8 nm), 75% quantile (78.2 nm), and upper whisker (132.9 nm). **(c)** Distribution of median lateral and axial localization precisions in whole chromosomes and within the pericentromeric regions labeled by Topo IIgp13 (n=10) and Topo IIrb12 (n=12). Numbers below the boxplots indicate the mean ± standard deviation. **(d)** Distribution of lateral and axial localization precisions (boxplots) of Topo II molecules labeled by gp13 and rb12 antibodies detected within the whole chromosomes and in their pericentromeric regions. These single measurements were used to calculate the averaged values shown in (c).

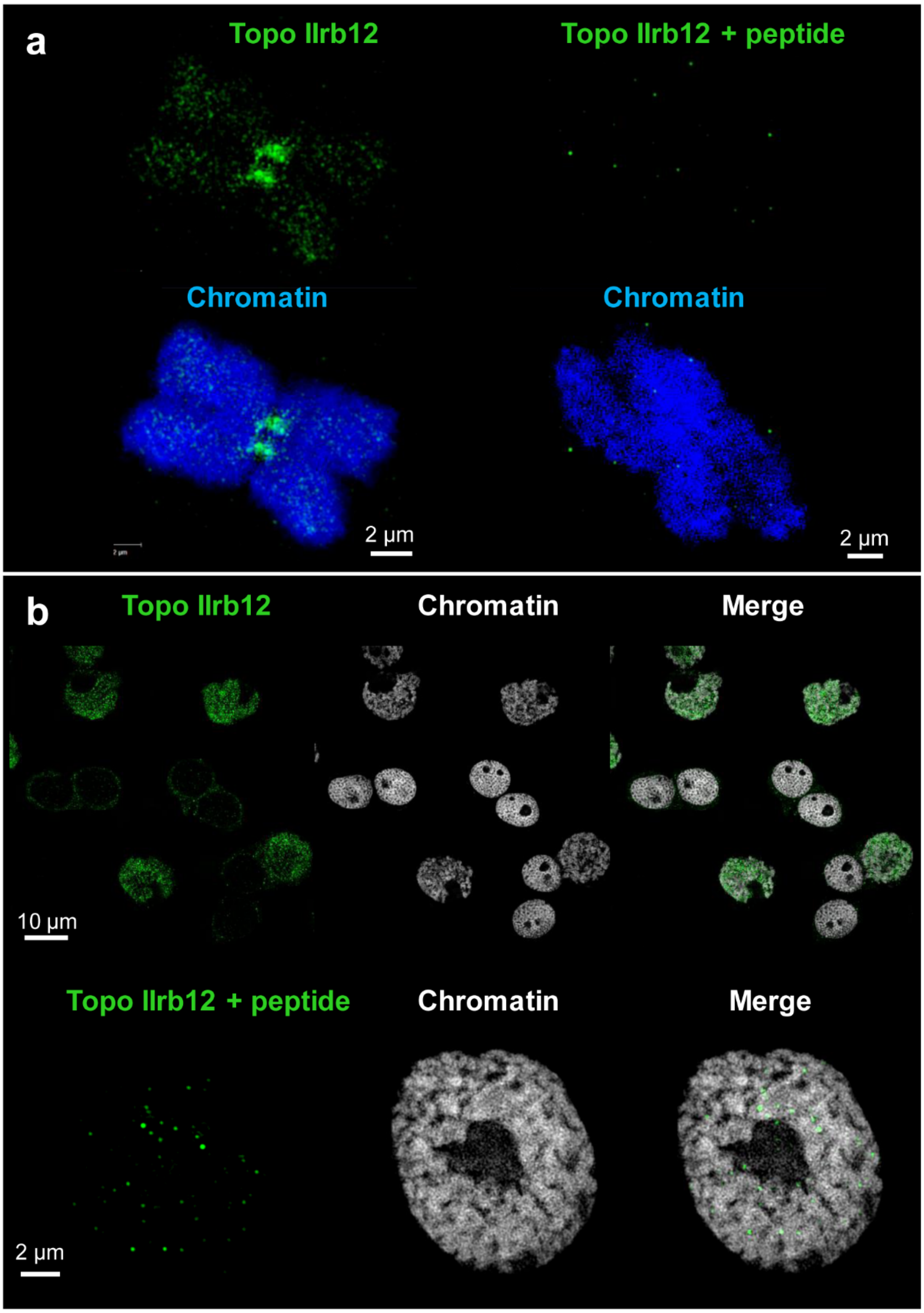

Figure S4. Topo IIrb12 antibody specificity proof by peptide competition. The antibodies become specifically sequestered by the corresponding peptide. (a) An isolated barley chromosome immunolabeled with Topo IIrb12 shows fluorescence signals at centromeres and chromosome arms (left). The addition of the peptides, in excess, to the Topo IIrb12 antibodies, resulted in a complete competition. No fluorescence signals were detected along the chromosome (right). (b) The similar behavior was observed at a population of barley interphase nuclei present in anther tissues. The Topo IIrb12 immunolabeling (left) and global chromatin staining of nuclei by DAPI (middle) is shown merged rightward.

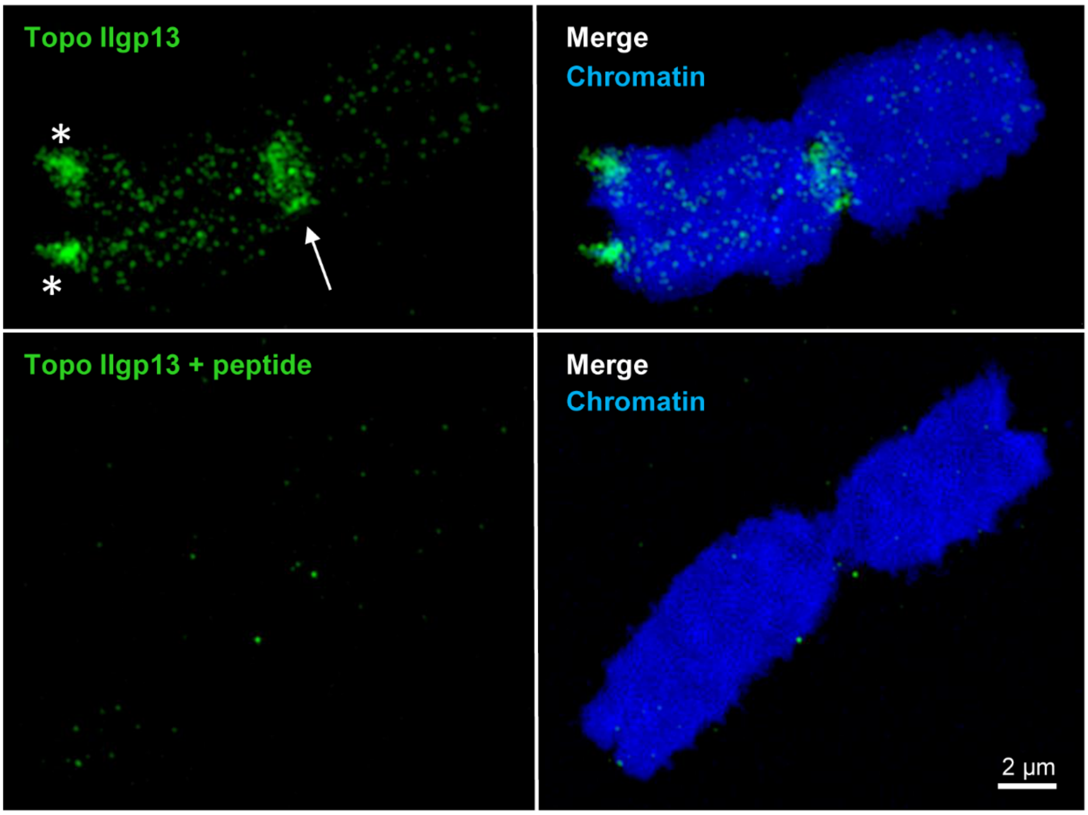

Figure S5. Topo IIgp13 antibody specificity proof by peptide competition. The antibodies become specifically sequestered by the corresponding peptide. An isolated barley chromosome immunolabeled with Topo IIgp13 shows fluorescence signals at centromeres (arrow), telomeres (asterisks), and chromosome arms (top). The addition of the peptides, in excess (1:100), to the antibodies, resulted in a complete competition. No fluorescence signals were detected along the chromosome (bottom).

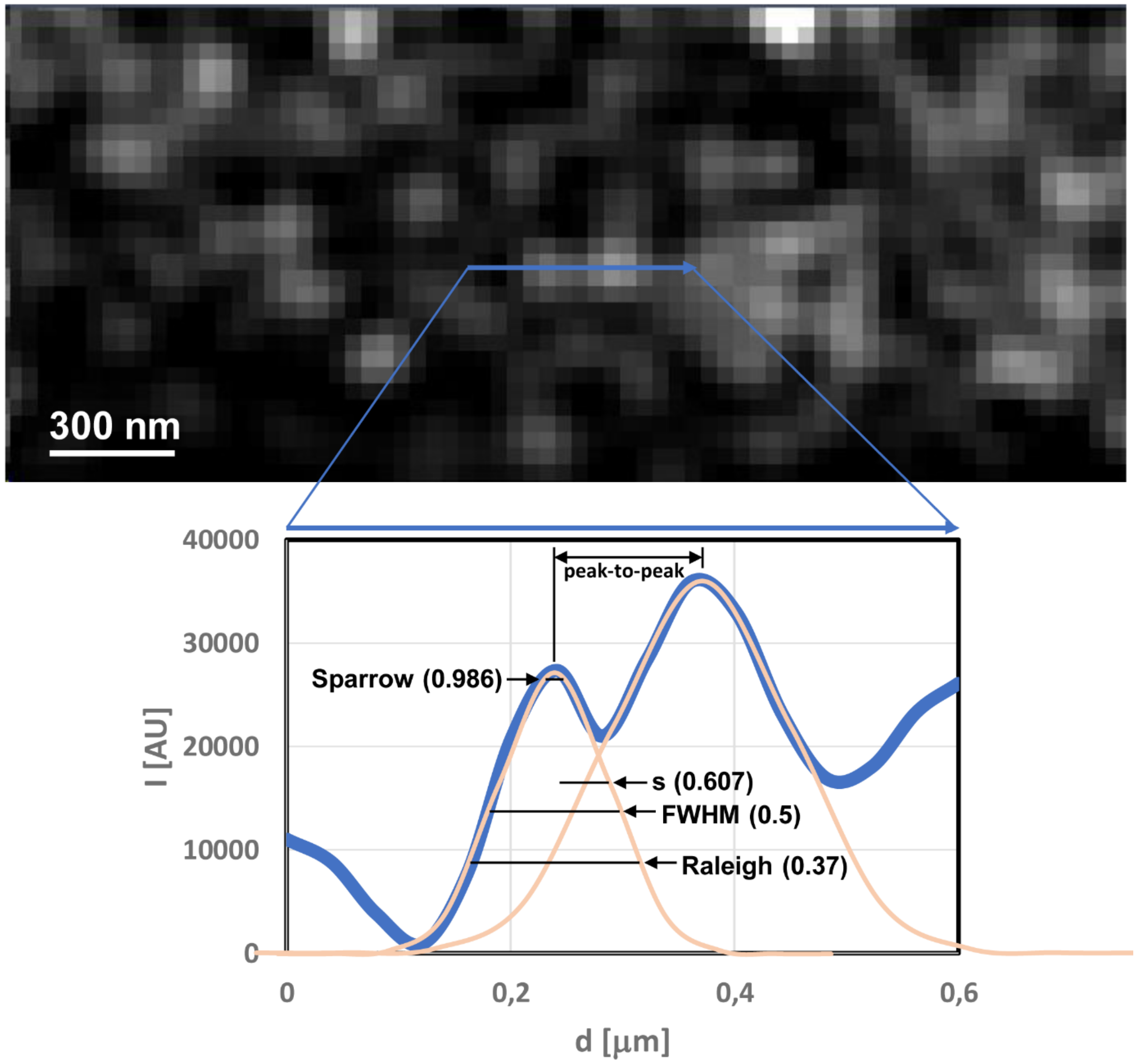

Figure S6. Resolution determination via profiles. The top image shows a Topo II-Alexa488-labeled chromatin region of a barley metaphase chromosomes visualized by SIM. The profile (blue arrow) is shown as a plot (blue line) in the lower panel with distances (d) given in µm and the intensity (I) in arbitrary units (AU, grey values). Peaks were approximated by Gauss functions (orange lines). For the Gauss function with the lower intensity peak (left) Sparrow width, standard deviation (s), full width at half maximum (FWHM) and the Raleigh width are indicated, along with the fraction of the maximum intensity. The dip between the peaks is low enough that the Sparrow criterion applies. Hence the peak-to-peak distance will give the resolution according to Sparrow. Note that in this case, the Raleigh criterion and the FWHM do not apply as the Gauss functions are too close to each other

Movie 1: 14 slices (Z-distance 100 nm) of a SIM image stack of a barley chromosome shown in Figure 4.
https://files.ipk-gatersleben.de/file/jznd17jJBUS7xoye/oqKdybQ73yBaEZeJ/Movie%201.avi

Movie 2: 3D rendering of 14 slices of a SIM image stack of a barley chromosome shown in Figure 4.
https://files.ipk-gatersleben.de/file/jznd17jJBUS7xoye/lsD542wzoV2gfRSk/Movie%202.mp4

Movie 3: 71 slices (Z-distance 20 nm) of a PALM image stack of a barley chromosome shown in Figure 4.

https://files.ipk-gatersleben.de/file/jznd17jJBUS7xoye/ZH9Szo8fq3fc6Kpc/Movie%204.mp4

Movie 4: 3D rendering of 71 slices of a PALM image stack of a barley chromosome shown in Figure 4. The first part shows Gauss rendering, the second part surface rendering of the Gauss signals.
https://files.ipk-gatersleben.de/file/kMwreDAnmmgWRPvy/odOg7djzTYF76Nst/Movie%203.avi
